# *In vitro* and *in vivo* anthelmintic activity of *Artemisia absinthium* against gastrointestinal nematodes of free-grazing goats from Ayacucho, Peru

**DOI:** 10.5455/javar.2025.l960

**Published:** 2025-09-22

**Authors:** Vania Flores-Prado, David Godoy-Padilla, Milagros Limaymanta-Zavala, Juancarlos Cruz-Luis, Daniel Zárate-Rendón

**Affiliations:** 1 Laboratorio de Parasitología, Departamento Académico de Nutrición, Facultad de Zootecnia, Universidad Nacional Agraria La Molina (UNALM), Lima, Peru; 2 Dirección de Supervisión y Monitoreo en las Estaciones Experimentales Agrarias, Instituto Nacional de Innovación Agraria (INIA), Lima, Peru

**Keywords:** *Artemisia absinthium*, anthelmintic resistance, gastrointestinal nematodes, goats, *Trichostrongylus* spp.

## Abstract

**Objective:**

The study assessed the anthelmintic activity of *Artemisia absinthium* ethanolic extract for controlling gastrointestinal nematodes in goats, both *in vitro* using infective larvae and *in vivo* in naturally infected goats under free-grazing conditions in the region of Ayacucho, Peru.

**Materials and Methods:**

For the *in vitro *assay, a larval motility inhibition test was performed on *Trichostrongylus* spp. infective larvae from goats using three different dilutions: 150, 175, and 200 mg/ml of the plant extract. *In vivo* efficacy was evaluated through the fecal egg count reduction test (FECRT), which was applied to 15 naturally infected Creole goats in one community. Animals were randomly assigned to 3 experimental groups and treated orally with 600 mg/kg of the plant extract. Fecal samples were collected directly from the rectum on days 0 (pre-treatment) and 7 and 15 post-treatment for egg count analysis.

**Results:**

*In vitro* results showed the highest inhibition of larval motility (81.79%) and larvicidal efficacy (82.2%) at the highest extract concentration (200 mg/ml). However, the *in vivo* results indicated that *A*. *absinthium*, at the concentration used, did not exhibit any significant effect on the FECRT.

**Conclusion:**

Although *A*.* absinthium* showed promising *in vitro* anthelmintic effects, the extract failed to demonstrate significant efficacy *in vivo* at the tested dose. Additionally, continuous monitoring of drugs in the region of study is strongly recommended based on the results obtained for albendazole.

## Introduction

Gastrointestinal nematode (GIN) infections represent one of the most significant health problems in small ruminant production systems, causing considerable economic losses in sheep and goats worldwide [1]. Conventionally, the control of these parasitic infections has relied heavily on the routine administration of anthelmintic drugs. However, the repeated use of these compounds has led to the selection of resistant parasite strains, severely undermining the efficacy of current control strategies [2,3]. In goats, resistance to the most common anthelmintics, such as albendazole, has already been reported [4,5]. Because of this critical situation, it is necessary to research non-pharmacological alternative approaches to controlling GIN. The plants with phytochemical compounds that exert anthelmintic properties constitute a promising option for controlling GIN in goats [6].

There are several reports of *in vitro* assays for different plant extracts against various stages of nematodes from both monogastric and ruminant hosts [7]. In goats, there have been a number of studies using different plant species and associations of plant secondary metabolites with anthelmintic effects [8]. In México, for instance, *Phytolacca icosandra* was tested *in vivo* on goats artificially infected with *Haemonchus contortus*, showing a significant reduction in parasite burden following oral administration of 250 mg/kg body weight on two consecutive days [9]. Similarly, Extracts of *Punica granatum *L., *Uncaria guianensis,* and *Carapa guianensis* were tested *in vitro* against GI nematodes, such as *H. contortus*, showing a clear anthelmintic activity at the parasite’s different stages [10–12].

Among the variety of medicinal plants, *Artemisia absinthium *(locally known as “ajenjo” in Peru) has been commonly used for different therapeutic purposes. Several *in vitro* and *in vivo* studies with this plant have shown its anthelmintic efficacy against several parasites, including GIN infecting small ruminants, swine, and equines [13–15]. The present study assessed the anthelmintic activity of *A*. *absinthium* ethanolic extract for controlling GIN in goats, both *in vitro* using infective larvae and *in vivo* in naturally infected goats under free-grazing conditions in the region of Ayacucho, Peru.

## Materials and Methods

### Ethical approval

All *in vivo* assay procedures were conducted in accordance with Peruvian National Law No. 30407, “Animal Protection and Welfare,” as well as international animal welfare standards. The animal trial was developed with the support of a veterinarian from the involved institution, who oversaw the dosing process. The assay was developed with the consent of the goat farmers, and it did not alter their usual management practices. The dosage was administered in a manner consistent with the routine use of veterinary medicines in livestock.

### Plant material

*Artemisia absinthium *specimens, collected in the region of Ancash (Northern, Perú), were purchased from a local market in Lima, Perú (11° 56’ 16.75’’ S 77° 03’ 56.70’’ O). The leaves were collected separately and subjected to drying in an oven at 50°C [16]. Dried leaves were ground and then stored until extraction.

### Plant extract

The *A*. *absinthium *leaf extract was prepared in ethanol. In brief, 175 gm of ground leaves were homogenized in 700 ml of 70% ethanol in a glass container [17,18]. This was allowed to macerate at ambient temperature for 48 h. Following maceration, the homogenate was filtered through cheesecloth. The filtered solution was evaporated at 45°C until a dried ethanolic extract was obtained. The dried extract was then transferred to a clean glass container and stored at 4°C [17,18].

### In vitro assays

***Trichostrongylus* spp. third-stage larvae (L3)**

Third-stage larvae (L3) from *Trichostrongylus *spp. were collected from goats with natural gastrointestinal infections. For this purpose, a pool of fecal material obtained from naturally infected goat herds was used to perform coprocultures. Then, larvae were recovered through a Baermann apparatus. A hundred percent of larvae were identified as *Trichostrongylus* spp. by morphological keys [19].

### In vitro anthelmintic activity assessment

A modified inhibition larval motility assay was performed using *Trichostrongylus* spp. L3s [11]. In brief, larvae were washed and suspended in distilled water. The plant extract was used in three concentrations (150, 175, and 200 mg/ml), prepared by dilution in distilled water following the methodology outlined by Nsereko et al. [18]. Distilled water served as the negative control. Approximately 15 larvae were incubated at 37°C in each treatment and control. Larval motility/inactivity evaluations were made at 0, 1, 2, 4, and 8 h post-exposure under a dissecting microscope. At each evaluation, larvae were resuspended and vortexed to stimulate them to move.

The mortality percentage was calculated using the following formula [20]:


$$\mathrm{Mortality}\;\mathrm{(\%)}\;=\;\frac{\mathrm{No}.\;\mathrm{of}\;\mathrm{paralyzed}\;/\;\mathrm{dead}\;\mathrm{larvae}}{\mathrm{No}.\;\mathrm{of}\;\mathrm{active}\;\mathrm{larvae}\;+\;\mathrm{No}.\;\mathrm{of}\;\mathrm{paralyzed}\;\mathrm{larvae}}\;\times \;100$$

The motility inhibition percentage was calculated following the formula [11]:


$$\mathrm{Inhibition}\;\mathrm{(\%)}\;=\;\frac{\mathrm{No}.\;\mathrm{of}\;\mathrm{active}\;\mathrm{larvae}\;\mathrm{negative}\;\mathrm{control}\;/\;\mathrm{No}.\;\mathrm{of}\;\mathrm{active}\;\mathrm{larvae}\;\mathrm{treatment}\;\mathrm{group}}{\mathrm{No}.\;\mathrm{of}\;\mathrm{active}\;\mathrm{larvae}}\;\times \;100$$

### In vivo assay

**Study area**

**Figure 1.**
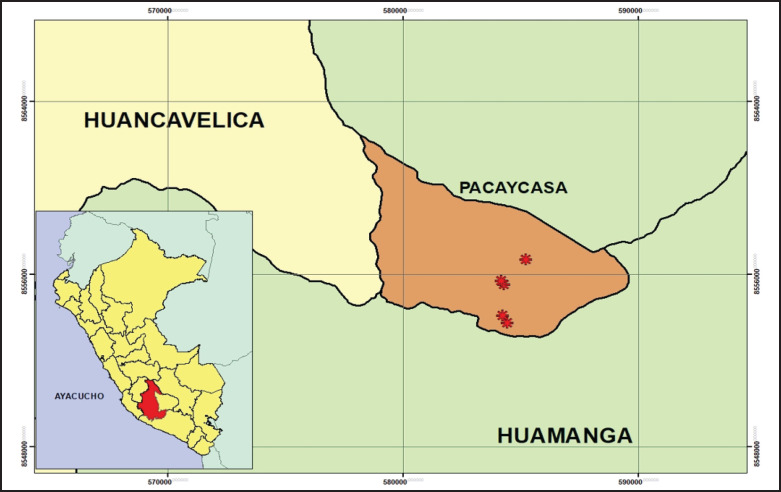
Study area of the Ayacucho Region, Peru, showing collecting sites from the Pacaycasa District, Huamanga Province.

The study was performed during the rainy season, specifically April and May 2023, in the District of Pacaycasa, located in the Huamanga Province, region of Ayacucho (Fig. 1). This location is at 2,500 m above sea level (masl) with maximum daily temperatures of 21°C and minimum temperatures of 10°C.

**Animals and experimental design**

Fifteen creole goats of > 6 months naturally infected with GIN were used for the *in vivo *test. According to their parasitic burden, expressed as eggs per gram (EPG), the animals were randomly allocated into three experimental groups. Each group was assigned to a different treatment, administered orally. Group 1 (*n =* 5) received the plant extract at a dose of 600 mg/kg body weight, administered once per day for three consecutive days. Group 2 (*n =* 6) was treated with a single albendazole dose (10 mg/kg). Group 3 (*n =* 4) received no treatment (negative control).

**Fecal egg count reduction test (FECRT)**

To evaluate the anthelmintic efficacy of the plant extract, FECRT was conducted. Fecal samples from animals in each treatment group were collected at 0, 7, and 15 post-treatment days. Samples were transported in cool boxes to the Parasitology Laboratory, Facultad de Zootecnia, Universidad Nacional Agraria La Molina, where they were refrigerated until processing. A 3-chamber McMaster slide was used to perform the fecal egg counts.

### Data analysis

The efficacy of each treatment was determined on days 15 and 30 post-treatment, employing the FECRT under the guidelines established by the World Association for the Advancement of Veterinary Parasitology (WAAVP) using the FECRT formula, as follows:


%Reduction=AverageepgDay0−AverageepgDay15/30AverageepgDay0×100

The 95% confidence intervals (CI) were estimated using the bootstrapping method. Larval motility inhibition and mortality assay results were expressed through arithmetic mean and standard error of the mean (SEM). Statistical differences among treatments in these assays were evaluated through analysis of variance, followed by Tukey’s test. The arithmetic mean of the FECRT results was analyzed by the Wilcoxon Test to compare the treatments’ efficacy. All statistical procedures were performed in R software (version 4.1.2; R Core Team, 2013, Vienna, Austria).

## Results

### In vitro anthelmintic activity

*Trichostrongylus *spp. larval mortality percentages are shown in Table 1. The most pronounced larvicidal effect (82.2%) was recorded at 8 h in the highest extract concentration (200 mg/ml).

**Table 1.** *Artemisia absinthium* ethanolic extract effects on *Trichostrongylus *spp. Third-stage larvae (L3) were obtained from naturally infected goats.

Treatments	Arithmetic media of larvae exhibiting motility after treatment exposure				
	0 h	1 h	2 h	4 h	8 h
Plant extract 150 mg/ml	15 ± 0.00	11.33 ± 0.88 (24.47)	9.67 ± 0.88 (35.53)	8 ± 1 (46.6)	5.67 ± 1.20 (62.2)
Plant extract 175 mg/ml	15 ± 0.00	8.67 ± 0.67 (42.2)	7.67 ± 0.33 (48.87)	5.67 ± 0.33 (62.2)	4.33 ± 0.88 (71.13)
Plant extract 200 mg/ml	15 ± 0.00	7.33 ± 0.58 (51.13)	5.67 ± 0.88 (62.2)	4.33 ± 0.33 (71.13)	2.67 ± 0.33 (82.2)
Control	15 ± 0.00	15 ± 0.00	15 ± 0.00	14.67 ± 0.33	14.67 ± 0.33

Figures in parentheses indicate larval mortality (mortality%); SE, standard error of mean.

**Figure 2.**
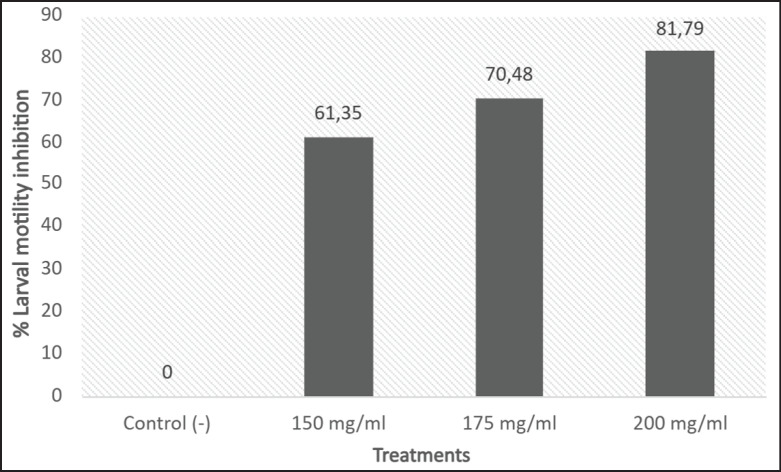
*Artemisia absinthium* extract anthelmintic effect at three different dilutions in a larval motility inhibition test using *Trichostrongylus *spp. third-stage larvae (L3) from goats expressed in % (*p* < 0.0005).

The *A*. *absinthium* extract at 3 dilutions showed a significant anthelmintic effect (*p* < 0.0005) on L3, which presented paralysis and/or mortality. At 8 h post-exposure, the plant extract induced a general larval motility inhibition percentage of 61.35%, 70.48%, and 81.79% at concentrations of 150 mg/ml, 175 mg/ml, and 200 mg/ml, respectively (Fig. 2).

Larval motility decreased gradually during the assay; significant differences were found at concentrations of 150 and 200 mg/ml from 1 through 4 h post-treatment. However, by the 8 h mark, no significant differences were detected among the tested concentrations.

### Fecal egg count reduction test

Administration of the ethanolic extract of *A. absinthium* (at a dose of 600 mg/kg) showed no significant anthelmintic activity when measured by a FECRT in naturally infected goats (Table 2). Albendazole showed optimal efficacy at 7 and 15 days post-treatment, 94.83% and 91.38%, respectively.

## Discussion

The *in vitro* assays showed higher larval motility inhibition (81.79%) at the highest extract concentration (200 mg/ml). These results confirm previous similar studies, such as the report made by Tariq et al. [17], who found significant larval inhibition of *Haemonchus contortus* larvae from sheep following exposure to a 25 mg/ml ethanolic preparation of *A*.* absinthium*. According to the WAAVP, for an *in vitro* evaluation of an anthelmintic compound, a larval motility inhibition higher than 90% is considered optimal efficacy, while a percentage range from 80% to 90% is considered to be moderate efficacy [21]. Therefore, our results would indicate that the *A*. *absinthium* ethanolic extract at a concentration of 200 mg/ml has moderate efficacy as an anthelmintic agent. However, these parameters are meant for commercial anthelmintic drugs and not for plant extracts; therefore, our results should be interpreted considering this. In this context, a moderate efficacy could be suitable for an ethnopharmacological approach for GIN control.

**Table 2.** Average eggs per gram (epg) for experimental groups and efficacy (%) at 7 and 15 post-treatment days of an *Artemisia absinthium* ethanolic extract and albendazole in a fecal egg count reduction test (FECRT).

Group	Dose (kg/kg)	Average eggs per gram (epg)		
		Day 0	Day 7	Day 15
*A*. *absinthium*	600 mg	396.8 ± 96.32	496 ± 114.822 (−25)	390.4 ± 184.285 (1.61)
Albendazole	10 mg	162.69 ± 43.23	6.86 ± 7.41 (94.83)	11.43 ± 9.77 (91.38)
Control	-	144 ± 55.81	244 ± 38.85 (−69.44)	388 ± 111.31 (−169.44)

Figures in parentheses indicate mean egg count percent reduction (FECR%); SE, standard error of mean.

The observed *in vitro* anthelmintic efficacy of the plant extract is likely attributable to the diverse array of phytochemical compounds in *A*. *absinthium, *whose nature and concentration vary according to the geographic region and different environmental conditions. For example, in Poland, the major components found in this species were sabinyl acetate, chrysanthenyl acetate, α-thujone, and β-thujone [22]. However, essential oil analysis from specimens collected in Turkey revealed camphor and 1,8-cineole [23]. Many authors believe that the anthelmintic activity of *A*. *absinthium* comes from the flavonoids quercetin and apigenin [24,25], and the content of these metabolites may be decisive in the level of anthelmintic efficacy, which in the present study could have been considerable.

Under *in vivo *conditions, the *A*. *absinthium* ethanolic extract (dose of 600 mg/kg) did not exhibit any anthelmintic activity against adult GIN in naturally free-grazing infected goats. This outcome highlights the fact that *in vitro* effects do not correlate with *in vivo* results. Several factors could explain these results, such as the bioavailability and pharmacology of phytochemical compounds in the host, the metabolism of bioactive herbal substances and their interaction with the ruminal microbiota, and experimental conditions [26]. Goats have lower absorption and greater metabolization of anthelmintics compared to other ruminants [27].

The plant extract concentration we used in the FECRT was even higher than the one applied in the *in vitro* assay; however, this concentration was not enough to exert a significant reduction in fecal eggs in infected goats [4]. Despite this outcome, a significant anthelmintic effect evidenced in other *in vivo* studies using other hosts cannot entirely be discarded [13].

Baudinette et al. [4] state that one contributing factor to the development of anthelmintic resistance in goats could be the result of the rapid metabolism of drugs exerted by these animals, which, in turn, significantly reduces the dose that reaches the parasite, increasing the chances of survival and development of resistance. On the other hand, albendazole showed acceptable anthelmintic efficacy on day 7, although, according to WAAVP standards (FECRT efficacy less than 95%) [28], this drug would be at the limit to continue being considered effective against GIN in goats from the Pacaycasa district of Ayacucho, Peru. However, on both days 7 and 15, lower limits of less than 95% were obtained. Therefore, it is considered that there is a significant reduction in efficacy, which would indicate suspicion of resistance. These results would suggest continuing to monitor the therapeutic efficacy of this drug and other pharmacological alternatives in this region.

Additional research is necessary to determine the viability of *A*. *absinthium* extract as a sustainable alternative approach for managing GIN goats. Moreover, continuous monitoring of drugs in the region of study is strongly recommended based on the results obtained for albendazole.

## Conclusion

This study contributes additional evidence regarding the* in vitro* anthelmintic activity of *A*. *absinthium* ethanolic extract against GIN larvae; however, its lack of observable efficacy under natural *in vivo *conditions in goats from Ayacucho, Peru, suggests that further investigation is warranted. Specifically, *in vivo* trials involving higher doses (> 600 mg) and at different post-treatment times should be performed before drawing definitive conclusions on its potential use in goat herds.

## List of abbreviations

ANOVA, Analysis of variance; CI, Confidence intervals; EPG, Egg per gram; FECRT, Fecal egg count reduction test; GIN, Gastrointestinal nematodes; L3, Third-stage larvae; SEM, Standard error of the mean; WAAVP, World Association for the Advancement of Veterinary Parasitology; h, hour; m, meter; gm, grams; ml, milliliter; mg, milligrams.
